# Combination of Structure Databases, In Silico Fragmentation, and MS/MS Libraries for Untargeted Screening of Non-Volatile Migrants from Recycled High-Density Polyethylene Milk Bottles

**DOI:** 10.1021/acs.analchem.2c05389

**Published:** 2023-06-01

**Authors:** Qi-Zhi Su, Paula Vera, Cristina Nerín

**Affiliations:** † Department of Analytical Chemistry, GUIA Group, I3A, EINA, University of Zaragoza, María de Luna 3, 50018 Zaragoza, Spain; ‡ National Reference Laboratory for Food Contact Material (Guangdong), Guangzhou Customs Technology Center, Guangzhou 510075, China

## Abstract

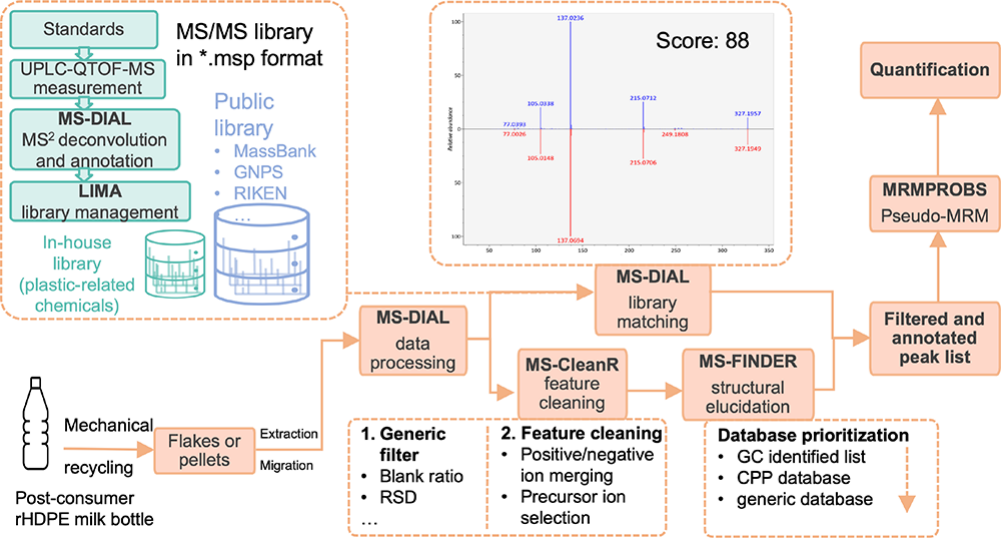

Chemical contamination is one of the major obstacles for mechanical recycling of plastics. In this article, we built and open-sourced an in-house MS/MS library containing more than 500 plastic-related chemicals and developed *mspcompiler,* an R package, for the compilation of various libraries. We then proposed a workflow to process untargeted screening data acquired by liquid chromatography high-resolution mass spectrometry. These tools were subsequently employed to data originating from recycled high-density polyethylene (rHDPE) obtained from milk bottles. A total of 83 compounds were identified, with 66 easily annotated by making use of our in-house MS/MS libraries and the *mspcompiler* R package. In silico fragmentation combined with data obtained from gas chromatography–mass spectrometry and lists of chemicals related to plastics were used to identify those remaining unknown. A pseudo-multiple reaction monitoring method was also applied to sensitively target and screen the identified chemicals in the samples. Quantification results demonstrated that a good sorting of postconsumer materials and a better recycling technology may be necessary for food contact applications. Removal or reduction of non-volatile substances, such as octocrylene and 2-ethylhexyl-4-methoxycinnamate, is still challenging but vital for the safe use of rHDPE as food contact materials.

Recycling of food contact plastics attracts increasing interest recently under the context of circular economy. According to EU regulation 10/2011,^1^ recycled materials may not have a negative effect on consumer health. Several studies have been conducted over the last few decades to investigate migratable substances from rHDPE,^2−7^ including the examination of odorants^8−11^ and volatile substances.^12^ While these studies have primarily focused on volatile substances and utilized well-developed GC–MS and commercial libraries, to the best of our knowledge, non-targeted screening of non-volatile compounds in recycled polyolefins remains largely unaddressed. This type of screening is more sophisticated and requires the use of expensive high-resolution mass spectrometry (HRMS) as well as specialized expertise.

Identification of non-volatile substances in FCM employing HRMS together with vendor software has been well documented.^13−15^ In the last decade, various open-source tools like XCMS,^16^ MZmine 2,^17^ MS-DIAL,^18^ and in silico fragmentation tools, e.g., MetFrag,^19^ MS-FINDER,^20^ and SIRIUS 4,^21^ have been developed to facilitate and improve the handling of HRMS data in the metabolomics community. Nonetheless, they are, in principle, applicable to the identification of any small molecule.^22^ These tools are of major interest for novelty, easy accessibility, and reproducibility.^23^ In contrast to vendor software, they are able to leverage publicly available MS/MS libraries like MassBank, RIKEN, and GNPS libraries and can connect to advanced tools, for example, CAMERA,^24^ MS-CleanR,^25^ and CliqueMS^26^ for feature (mass–retention time pair) cleaning.

This study aims to comprehensively analyze non-volatile migrants coming from rHDPE milk bottles by ultrahigh-performance liquid chromatography coupled to quadrupole time-of-flight mass spectrometry (UPLC-QTOF-MS) and advanced data processing workflow. An R package, namely, *mspcompiler*, was developed to compile various publicly accessible and in-house MS/MS libraries. The library was then utilized for identification in MS-DIAL. MS-CleanR was employed to clean up redundant features including a number of adducts and in-source fragments. An in silico fragmentation tool (MS-FINDER) was finally applied to identify the remaining unknowns, taking advantage of the list of chemicals previously identified in the same set of samples by GC–MS and a list of chemicals associated with plastic packaging compiled by Groh et al^27^ After the chemicals were annotated, pseudo-multiple reaction monitoring (pseudo-MRM) using parent–product ion pairs exported from MS-DIAL was employed as a sensitive targeted analysis to determine the presence of each identified substance in the samples using the MRMPROBS program. Finally, the concentrations of the annotated compounds in the simulants were quantified.

## Materials and Methods

Descriptions about the samples have been detailed in our previous article.^12^ In brief, rHDPE milk bottles in flake (F) and pellet (P) forms were supplied by three Spanish companies. Samples F1.1, F1.2, F1.3, F1.3′, P1.1, P1.2, P1.3, and P1.3′ were from company 1; samples F2.1, F2.2, F2.3, F2.4, F2.5, F2.6, P2.1, P2.2, and P2.3 were from company 2; samples F3.1, F3.2, P3.1, and P3.2 were from company 3.

### Solid–Liquid Extraction

Samples were first ground into fine powders, weighed (1.00 g), and extracted 3 times with 5 mL of dichloromethane under an ultrasonic bath for 1 h. Subsequently, the extracts were merged and evaporated to dryness by a gentle flow of nitrogen at 40 °C. Then, the extract was re-dissolved with 0.4 mL of methanol for 5 min under ultrasonication and 30 s of vortex mixing and filtered (0.2 μm Acodisc GHP syringe filter) prior to UPLC-QTOF-MS analysis. Samples and procedural blanks were simultaneously prepared in triplicate. Quality control (QC) sample was pooled from filtered extracts (50 μL from each sample).

### Migration Test

Migration test was carried out based on the regulation EU 10/2011, which has been well-documented in our previous study.^12^ Samples as well as procedural blanks were simultaneously prepared in duplicate.

### UPLC-QTOF-MS Analysis

A Waters Acquity UPLC was used with an Atlantis premier BEH C18 AX column (2.1 mm × 100 mm, 1.7 μm particle size) at a column temperature of 40 °C and a flow rate of 0.3 mL/min. The mobile phase consisted of water (A) and methanol (B) with 0.1% formic acid in both positive and negative modes with a gradient elution over a 13 min run. The initial mobile phase was 95/5 A/B shifted to 100/0 A/B in 7 min, kept for 4 min, dropped to the initial phase in 0.1 min, and maintained for 1.9 min before the next injection. The injection volume was 10 μL. The UPLC and the QTOF-MS were interfaced by an electro spray ionization (ESI) probe. Low energy (6 V) and ramp high energy (10–30 V) were used for data acquisition (MS^E^ mod) scanning from 50 to 1200 Da. Leucine enkephalin was employed for online mass correction. Test-mix from Waters was injected every 20 injections to ensure accuracy of the data.

The data was processed by MS-DIAL (version 4.38)^18^ with a mass tolerance of 0.01 and 0.025 Da for MS1 and MS2, respectively. Adducts in negative mode were [M-H]^−^, [M + FA-H^]–^, [M + Hac-H]^−^, [2 M-H]^−^, [2 M + FA-H]^−^, and [2 M + Hac-H]^−^, and those in positive mode were [M + H]^+^, [M + NH_4_]^+^, [M + Na]^+^, [M + K]^+^, [2 M + H]^+^, [2 M + NH_4_]^+^, [2 M + Na]^+^, and [2 M + K]^+^; the minimum peak height was 3000, and the identification score cut-off was 80%. Only peaks that had sample max/blank ratios higher than 5 were kept.

### In-House MS/MS Library and *mspcompiler* R Package

An in-house MS/MS library containing 449 and 172 plastic-related chemicals in the positive and negative modes, respectively, was built following a strategy proposed by Tada et al.,^28^ which is available at https://github.com/QizhiSu/MS-libraries. In addition, we developed a R package, *mspcompiler*, to clean and compile various MS/MS libraries for identification in MS-DIAL. The details are available in Appendix A.

### MS-CleanR Feature Cleaning and Structural Elucidation by MS-FINDER

Feature tables exported from MS-DIAL were cleaned by removing those with blank/QC and relative standard deviations higher than 0.5 and 30, respectively, using the MS-CleanR package.^25^ Subsequently, the features were grouped into clusters using pre-calculated links, Pearson correlation, and other criteria. Clusters had to have a minimum Pearson correlation of 0.8 at a maximum *p*-value of 0.05 and maximum mass and retention time differences of 0.005 and 0.025, respectively, for positive/negative merging. It is worth noting that identical mobile phases should be used in both positive and negative modes to ensure the correlation of peaks as the pH value of mobile phases is well known to have great impact on the retention behavior of certain compounds. For subsequent structural elucidation in MS-FINDER,^20^ both the most intense and most connected features were kept. In addition, features that did not have representative MS/MS spectra (see example in Figure A1) were eliminated as well since they are meaningless for structural elucidation.

In MS-FINDER, the MS1 and MS2 tolerances were 0.005 and 0.025 Da, respectively, considering elements C, H, O, N, P, S, F, and Cl. Three structure databases were applied for the identification, namely, volatile and semi-volatile substances identified by GC–MS in the same set of samples in our previous study (volDB),^12^ chemicals associated with plastic packaging (cppDB) compiled by Groh K. J. etc.,^27^ and a generic database (genDB) integrated in MS-FINDER. The genDB includes only FoodDB (Food), PlantCyc (Plant), T3DB (Toxin), STOFF (Environment), NPA (Natural Products Atlas), KNApSAcK (Natural Product), NANPDB (Natural Product), and UNPD (Natural Product) because they could be contaminants in recycled plastics. The weights given to the three databases in MS-CleanR were 2, 1.5, and 1, respectively. Manual inspection of the MS/MS spectra was conducted to excluded compounds that did not have representative spectra as demonstrated in Figure A1 since most of the fragments were simply noise and meaningless for subsequent elucidation. The identification flow chart is shown in Figure A2.

### Pseudo-Multiple Reaction Monitoring by MRMPROBS

MRMPROBS is an open-source software designed for targeted metabolomics, providing automated posterior probabilistic data assessment to replace manual methods.^29^ In this study, MRMPROBS was used as a pseudo-MRM approach to detect identified compound in each sample. The top 5 product ions were exported as a MRMPROBS library which was then used for MRM analysis directly on a DIA data set. Unlike conventional MRM, no re-acquisition of MRM data is required. Parameters used included a smoothing level of 1, a minimum peak width of 5, and a minimum peak height of 100. Retention time tolerance, amplitude tolerance, and minimum posterior score were 0.1 min, 15%, and 60%, respectively. A peak area at least 3 times higher than the blanks was required for a compound to be considered present in a sample.

## Results and Discussion

### Identification by Matching against Libraries

As shown in Table 1, there were 66 compounds identified either in the extracts or migrates by library search. Although some compounds had low scores, they were still included because their scores were negatively affected by the large number of noisy signal present in the spectra. For instance, *N*-[3-(dimethylamino)propyl]dodecanamide and dimethyldiben-zylidene sorbitol had low scores (Figure A3), but they were confirmed by standards using retention time and exact mass of the precursor ion and the major fragments. The compiled library facilitated the identification of several pesticides (e.g., propanil and pyrimethanil) and plastic-related chemicals (e.g., caprolactam and Irgafos 168) by library matching.

**Table 1:** Migrants Identified in 95% Ethanol or 3% Acetic Acid Food Simulants^a^

no.	*R*_t_	precursor	adduct	name	CAS	formula	fill (%)	matrix	score	lev	remark
1	0.83	341.1081	[M-H]^−^	sucrose	57-50-1	C_12_H_22_O_11_	14.3	95EtOH	Lmatch (86)	I	
2	0.95	232.1456	[M + H]^+^	**aminophenazone**	58-15-1	C_13_H_17_N_3_O	9.5	95EtOH	genDB (6.2)	IV	drug
3	1.28	124.0759	[M + H]^+^	***o*-anisidine**	90-04-0	C_7_H_9_NO	14.3	3HAC	Lmatch (86)	V	CMR; SVHC
4	1.65	158.0972	[M + H]^+^	quinoline, 2,7-dimethyl-	93-37-8	C_11_H_11_N	28.6	3HAC	volDB (5)	IV	
5	1.77	122.0967	[M + H]^+^	**2,4-dimethylbenzenamine**	95-68-1	C_8_H_11_N	42.9	3HAC	Lmatch (88)	II	intermediate
6	2.61	202.0432	[M + H]^+^	**thiabendazole**	148-79-8	C_10_H_7_N_3_S	9.5	extract	Lmatch (81)	IV	drug
7	3.11	114.0915	[M + H]^+^	**caprolactam**	105-60-2	C_6_H_11_NO	23.8	3HAC	Lmatch (86)	II	nylon 6 monomer
8	3.46	195.0885	[M + H]^+^	**caffeine**	58-08-2	C_8_H_10_N_4_O_2_	47.6	extract	Lmatch (87)	IV	
9	3.92	142.0427	[M + H]^+^	3-chloro-*o*-toluidine	87-60-5	C_7_H_8_ClN	14.3	3HAC	cppDB (5.4)	IV	intermediate
10	4.78	274.2754	[M + H]^+^	***N*,*N*-bis(2-hydroxyethyl)dodecylamine**	1541-67-9	C_16_H_35_NO_2_	61.9	3HAC	Lmatch (97)	II	
11	4.83	214.2535	[M + H]^+^	*N*,*N*-dimethyldodecylamine	112-18-5	C_14_H_31_N	100	3HAC	cppDB (6.2)	II	antistatic
12	4.83	200.2371	[M + H]^+^	*N*-methyldodecylamine	7311-30-0	C_13_H_29_N	90.5	extract	Lmatch (87)	IV	
13	4.90	285.2917	[M + H]^+^	***N*-[3-(dimethylamino)propyl]dodecanamide**	3179-80-4	C_17_H_36_N_2_O	33.3	3HAC	Lmatch (72)	IV	antistatic
14	5.02	230.2482	[M + H]^+^	lauramine oxide	1643-20-5	C_14_H_31_NO	33.3	extract	Lmatch (92)	IV	surfactants
15	5.02	150.0912	[M + H]^+^	*N*-(2,4-dimethylphenyl)formamide	60397-77-5	C_9_H_11_NO	14.3	extract	Lmatch (84)	II	Insecticides
16	5.04	288.2896	[M + H]^+^	2-aminoheptadecane-1,3-diol		C_17_H_37_NO_2_	14.3	extract	Lmatch (87)	III	
17	5.33	202.0854	[M + H]^+^	simazine	122-34-9	C_7_H_12_ClN_5_	19	extract	Lmatch (90)	V	CMR; herbicide
18	5.34	242.2852	[M + H]^+^	***N*,*N*-dimethyltetradecylamine**	112-75-4	C_16_H_35_N	100	95EtOH	Lmatch (86)	II	antistatic
19	5.40	266.0975	[M + H]^+^	albendazole	54965-21-8	C_12_H_15_N_3_O_2_S	9.5	extract	Lmatch (80)	IV	drug
20	5.70	200.1191	[M + H]^+^	**pyrimethanil**	53112-28-0	C_12_H_13_N_3_	28.6	3HAC	Lmatch (92)	IV	fungicide
21	5.74	242.1439	[M + H]^+^	prometryn	7287-19-6	C_10_H_19_N_5_S	9.5	extract	Lmatch (88)	IV	herbicide
22	5.79	242.1435	[M + H]^+^	terbutryn	886-50-0	C_10_H_19_N_5_S	9.5	extract	Lmatch (78)	IV	herbicide
23	5.86	192.1384	[M + H]^+^	diethyltoluamide	134-62-3	C_12_H_17_NO	61.9	95EtOH	Lmatch (93)	II	insect repellent
24	5.88	270.3173	[M + H]^+^	***N*,*N*-dimethylhexadecylamine**	112-69-6	C_18_H_39_N	95.2	95EtOH		II	antistatic
25	5.95	312.3632	[M + H]^+^	***N*-methyldidecylamine**	7396-58-9	C_21_H_45_N	42.9	95EtOH	Lmatch (92)	II	intermediate
26	6.14	404.1255	[M + H]^+^	azoxystrobin	131860-33-8	C_22_H_17_N_3_O_5_	9.5	extract	Lmatch (89)	IV	fungicide
27	6.18	253.0307	[M + H]^+^	**3,3′-dichlorobenzidine**	91-94-1	C_12_H_10_Cl_2_N_2_	14.3	95EtOH	Lmatch (84)	V	intermediate
28	6.37	230.1168	[M + H]^+^	sebuthylazine	7286-69-3	C_9_H1_6_ClN_5_	28.6	extract	Lmatch (87)	IV	herbicides
29	6.43	215.9981	[M-H]^−^	**propanil**	709-98-8	C_9_H_9_Cl_2_NO	9.5	95EtOH	Lmatch (90)	IV	herbicides
30	6.43	182.0097	[M + H]^+^	2-(methylsulfanyl)-1,3-benzothiazole	615-22-5	C_8_H_7_NS_2_	9.5	extract	Lmatch (81)	IV	fungicides
31	6.48	194.1175	[M + H]^+^	**ethyl 4-(dimethylamino)benzoate**	10287-53-3	C_11_H_15_NO_2_	28.6	extract	Lmatch (91)	II	paint additives
32	6.71	332.0665	[M + H]^+^	piroxicam	36322-90-4	C_15_H_13_N_3_O_4_S	9.5	3HAC	genDB (5.9)	IV	drug
33	6.75	284.1428	[M + H]^+^	metolachlor	51218-45-2	C_15_H_22_ClNO_2_	9.5	extract	Lmatch (86)	IV	herbicide
34	6.76	415.2133	[M + H]^+^	**dimethyldibenzylidene sorbitol**	135861-56-2	C_24_H_30_O_6_	23.8	extract	Lmatch (64)	I	plastic additive
35	6.83	293.1735	[M-H]^−^	**3-(3,5-di-*tert*-butyl-1-hydroxy-4-oxocyclohexa-2,5-dien-1-yl)propanoic acid**		C_17_H_26_O_4_	100	3HAC	cppDB (6.4)	IV	NIAS
36	6.86	229.0862	[M + H]^+^	**oxybenzone**	131-57-7	C_14_H_12_O_3_	33.3	extract	Lmatch (85)	V	UV filter; EDC
37	6.91	198.1856	[M + H]^+^	**1-octylpyrrolidin-2-one**	2687-94-7	C_12_H_23_NO	23.8	95EtOH	cppDB (5.9)	IV	
38	6.94	308.1541	[M + H]^+^	**tebuconazole**	80443-41-0	C_16_H_22_ClN_3_O	9.5	95EtOH	Lmatch (83)	V	fungicides; CMR; EDC
39	6.97	305.1081	[M + H]^+^	diazinon	333-41-5	C_12_H_21_N_2_O_3_PS	9.5	extract	Lmatch (86)	IV	insecticide
40	7.00	342.078	[M + H]^+^	propiconazole	60207-90-1	C_15_H_17_Cl_2_N_3_O_2_	9.5	95EtOH	Lmatch (81)	V	fungicides; CMR
41	7.06	277.1817	[M + H]^+^	**7,9-di-*tert*-butyl-1-oxaspiro(4,5)deca-6,9-diene-2,8-dione**	82304-66-3	C_17_H_24_O_3_	47.6	95EtOH	Lmatch (83)	IV	NIAS
42	7.14	406.074	[M + H]^+^	difenoconazole	119446-68-3	C_19_H_17_Cl_2_N_3_O_3_	9.5	extract	Lmatch (85)	IV	fungicide
43	7.19	200.202	[M + H]^+^	lauramide	1120-16-7	C_12_H_25_NO	9.5	95EtOH	Lmatch (85)	IV	
44	7.20	310.2367	[M + Na]^+^	**lauric acid diethanolamide**	120-40-1	C_16_H_33_NO_3_	14.3	95EtOH	Lmatch (86)	II	antistatic
45	7.22	395.0808	[M + H]^+^	**diflufenican**	83164-33-4	C_19_H_11_F_5_N_2_O_2_	9.5	95EtOH	genDB (6.3)	IV	herbicides
46	7.24	220.1125	[M + H]^+^	*N*-phenyl-2-naphthylamine	135-88-6	C_16_H_13_N	9.5	95EtOH	volDB (5.4)	V	lubricant; CMR
47	7.30	383.2042	[M + Na]^+^	**tributyl citrate**	77-94-1	C_18_H_32_O_7_	85.7	95EtOH	cppDB (6.3)	IV	plasticizer
48	7.32	421.2326	[M + Na]^+^	tris(2-butoxyethyl) phosphate	78-51-3	C_18_H_39_O_7_P	42.9	extract	Lmatch (89)	IV	plasticizer
49	7.37	277.1812	[M-H]^−^	**3-(3,5-di-*tert*-butyl-4-hydroxyphenyl)propionic acid**	20170-32-5	C_17_H_26_O_3_	28.6	extract	Lmatch (88)	III	NIAS
50	7.40	273.1853	[M + H]^+^	galaxolidone		C_18_H_24_O_2_	28.6	extract	Lmatch (83)	IV	
51	7.47	179.0701	[M + H]^+^	3-methoxycinnamic acid	6099-04-3	C_10_H_10_O_3_	33.3	extract	Lmatch (86)	II	
52	7.48	599.1155	[M + Na]^+^	ethylene terephthalate cyclic trimer		C_30_H_24_O_12_	9.5	95EtOH	cppDB (5.5)	IV	PET oligomer
53	7.54	322.1454	[M + H]^+^	pyriproxyfen	95737-68-1	C_20_H_19_NO_3_	14.3	extract	Lmatch (84)	IV	insecticide
54	7.54	425.2149	[M + Na]^+^	tributyl acetylcitrate	77-90-7	C_20_H_34_O_8_	81	95EtOH	Lmatch (81)	I	plasticizer
55	7.55	199.1328	[M + H-H2O]^+^	sebacic acid monomethyl ester	818-88-2	C_11_H_20_O_4_	19	extract	Lmatch (80)	II	
56	7.63	255.1746	[M + H]^+^	4-methylbenzylidene camphor	36861-47-9	C_18_H_22_O	38.1	extract	Lmatch (84)	V	UV filter; EDC
57	7.67	295.2272	[M-H]^−^	9-hydroxy-10,12-octadecadienoic acid	10075-11-3	C_18_H_32_O_3_	76.2	extract	Lmatch (88)	III	
58	7.77	507.2737	[M + Na]^+^	**1,2,3-trideoxy-4,6:5,7-bis-*o*-[(4-propylphenyl)methylene]-nonitol (NX8000)**	882073-43-0	C_29_H_40_O_6_	33.3	extract	Lmatch (82)	II	plastic additive
59	7.88	384.1934	[M + Na]^+^	**octocrylene**	6197-30-4	C_24_H_27_NO_2_	100	95EtOH	Lmatch (90)	IV	UV filter
60	8.00	259.2065	[M + H]^+^	galaxolide 1	1222-05-5	C_18_H_26_O	28.6	95EtOH	volDB (5.8)	IV	
61	8.04	311.1645	[M + H]^+^	**avobenzone**	70356-09-1	C_20_H_22_O_3_	100	95EtOH	Lmatch (91)	IV	UV filter
62	8.06	291.1998	[M + H]^+^	**2-ethylhexyl 4-methoxycinnamate**	83834-59-7	C_18_H_26_O3	61.9	extract	Lmatch (83)	V	UV filter; EDC
63	8.13	443.3351	[M + H]^+^	1,2,3-propanetriol 1-stearate 2,3-bisacetate	33599-07-4	C_25_H_46_O_6_	100	95EtOH	cppDB (6)	II	
64	8.20	256.2647	[M + H]^+^	**palmitamide**	629-54-9	C_16_H_33_NO	42.9	extract	Lmatch (85)	IV	
65	8.47	381.298	[M + Na]^+^	**2-stearoylglycerol**	621-61-4	C_21_H_42_O_4_	76.2	95EtOH	Lmatch (92)	II	lubricant
66	8.50	327.1963	[M + H]^+^	**Chimassorb 81**	1843-05-6	C_21_H_26_O_3_	42.9	95EtOH	Lmatch (88)	II	UV absorber
67	8.52	437.3062	[M-H]^−^	2,4-di-*tert*-butylphenyl 3,5-di-tert-butyl-4-hydroxybenzoate (UV 120)	4221-80-1	C_29_H_42_O3	9.5	95EtOH	cppDB (5.7)	I	plastic additive
68	8.57	803.5445	[2 M + Na]^+^	**dioctyl phthalate**	117-84-0	C_24_H_38_O_4_	19	95EtOH	Lmatch (88)	V	plasticizer; EDC
69	8.63	393.2969	[M + Na]^+^	**bis(2-ethylhexyl) adipate**	103-23-1	C_22_H_42_O_4_	66.7	95EtOH	Lmatch (93)	II	plasticizer
70	8.81	255.2318	[M-H]^−^	**palmitic acid**	57-10-3	C_16_H_32_O_2_	71.4	95EtOH	Lmatch (91)	I	
71	8.81	360.324	[M + Na]^+^	**erucamide**	112-84-5	C_22_H_43_NO	61.9	95EtOH	Lmatch (85)	I	lubricant
72	8.86	647.4585	[M + H]^+^	**Irgafos 168**	31570-04-4	C_42_H_63_O_3_P	100	95EtOH	Lmatch (85)	I	antioxidant
73	8.86	281.2475	[M-H]^−^	**oleic acid**	112-80-1	C_18_H_34_O2	100	95EtOH	Lmatch (85)	I	
74	9.18	340.3574	[M + H]^+^	**docosanamide**	3061-75-4	C_22_H_45_NO	61.9	95EtOH	cppDB (5.9)	I	processing aid
75	9.25	469.3289	[M + Na]^+^	**diisodecyl phthalate**	89-16-7	C_28_H_46_O4	33.3	95EtOH	Lmatch (86)	V	plasticizer; EDC
76	9.36	1175.776	[M-H]^−^	**Irganox 1010**	6683-19-8	C_73_H_108_O_12_	100	95EtOH	Lmatch (89)	I	antioxidant
77	9.39	431.1784	[M + H]^+^	**2,5-bis(5-*tert*-butyl-benzoxazol-2-yl)thiophene**	7128-64-5	C_26_H_26_N_2_O_2_S	71.4	95EtOH	Lmatch (86)	III	plastic additive
78	9.61	199.0157	[M-H]^−^	(4-chloro-2-methylphenoxy)acetic acid	94-74-6	C_9_H_9_ClO_3_	9.5	3HAC	Lmatch (73)	IV	herbicides
79	10.24	663.454	[M + H]^+^	**oxidized Irgafos 168**	95906-11-9	C_42_H_63_O_4_P	100	95EtOH	Lmatch (98)	IV	NIAS
80	10.27	385.3471	[M + H]^+^	(+)-4-cholesten-3-one	601-57-0	C_27_H_44_O	90.5	extract	Lmatch (71)	IV	
81	11.44	553.4598	[M + Na]^+^	**Irganox 1076**	2082-79-3	C_35_H_62_O_3_	100	95EtOH	Lmatch (85)	II	antioxidant
82	11.60	591.4948	[M + Na]^+^	**glycerol dihexanoate**	502-52-3	C_35_H_68_O_5_	14.3	95EtOH	Lmatch (69)	II	emollient
83	11.73	533.529	[M + H]^+^	(*Z*)-octadec-9-enyl oleate	3687-45-4	C_36_H_68_O_2_	9.5	95EtOH	cppDB (5.1)	II	

a Chemicals in bold font were confirmed by a reference standard considering exact mass, retention time, and MS/MS spectra. The “fill (%)” column is the percentage of samples that detected the chemical in all samples (21 in total). The column “matrix” tells where the chemicals were identified. Some compounds were initially identified in the extracts by matching libraries, and their determination in the simulants were achieved by pseudo-MRM in MRMPROBS. Lmatch in the “score” column represents library match, and the number in the bracket were the scores (full mark 100) given by MS-DIAL, while others were the three structure databases that the compounds were finally identified, and the number in the bracket were the scores (full mark 10) given by MS-FINDER. The “lev” column gives the level of concern of the compounds based on the method previous proposed by our group.^30^ For detailed information, please refer to Appendix B. In the “remark” column, CMR represents carcinogenic, mutagenic, and reprotoxic chemicals, while EDC stands for endocrine-disrupting chemicals.

The retention time of some compounds differed between 95% ethanol and 3% acetic acid food simulants (Figure A4). This is expected as a reverse-phase column, and an initial mobile phase of 5% ethanol was used. For aqueous samples, such as 3% acetic acid, sample injection should not affect the mobile phase composition as the solvent and mobile phase are similar. However, for organic samples like 95% ethanol, the 10 μL injection volume takes time to mix with the mobile phase because the mobile phase contains more organic solvent, which elutes compounds faster and results in shorter retention time. In the field of food contact materials, various food simulants can be used, and compounds may have different retention time in these simulants, leading to misidentification. Additionally, caution should be exercised when using retention time information from MS/MS libraries or prediction models for identifying compounds in aqueous food simulants as organic solvents are commonly used to prepare standard solutions for building these databases.

### MS-CleanR Feature Cleaning and Structural Elucidation by MS-FINDER

Some features with high matching scores were removed by MS-CleanR as they were fragments rather than individual compounds. For instance, feature 106.0866 *m*/*z* at 7.20 min was identified as diethanolamine (Figure A5A), but it was actually an in-source fragment of the feature 288.2544 *m*/*z* at 7.20 min, which was identified as lauric acid diethanolamide (Figure A5B). After this, the remaining features were considered individual compounds and subjected to MS-FINDER for structural elucidation, which we will explain using chemical structure databases.

### Integrating Results from GC–MS

Using MS-FINDER to search the entire Pubchem can be slow and yield a large number of candidates, given that the database archives tens of millions of compounds. In certain types of samples, many compounds are suspect or already known to be present. This is why various structure databases are used for structural elucidation as they help in detecting suspect compounds in similar samples. While manually comparing fragmentation patterns to the ones shown in literature in similar samples is tedious and requires a lot of expertise, MS-FINDER can computationally fragment structures with the same molecular formula in selected databases and rank them based on factors like in silico fragmentation probability and frequency in the databases. Hence, using relevant structure databases can be beneficial in the investigation of the samples.

Over 200 compounds were previously identified in this set of samples using GC–MS by our group,^12^ but many of them can also be detected by liquid chromatography. If a compound has already been identified in GC–MS, it needs not be re-analyzed using UPLC-QTOF-MS. The challenge lies in identifying unknown peaks in UPLC-QTOF-MS that may correspond to a GC–MS-identified compound. To do this, one common approach is to calculate the exact mass and common adducts of the GC–MS identified compounds, like [M + H]^+^ and [M + Na]^+^ in positive mode and [M-H]^−^ in negative mode, and compare them to the UPLC-QTOF-MS peaks. While identical mass (within tolerance) may suggest the same compound, relying solely on exact mass may not be sufficient.

In this study, we proposed a more convenient and reliable way for correlating UPLC-QTOF-MS peaks to the GC–MS-identified compounds. This method involved creating a structure database of the GC–MS-identified chemicals and using it in MS-FINDER for structural elucidation. MS-FINDER rates molecular formulas based on mass errors, isotopic ratios, product ions, and neutral losses.^20^ It then predicts the MS/MS spectra of all chemicals with the same formula in the database and compares them to the acquired MS/MS spectra, a process known as precursor-oriented spectral search. By combining the exact mass and in silico MS/MS spectra, we can confidently match peaks to the corresponding compounds. For example, using the volDB, we matched the peak 214.2535 *m*/*z* at 4.826 min to *N*,*N*-dimethyldodecylamine (6 A), which was previously identified in this set of samples by GC–MS, and its distributions in GC–MS and UPLC-QTOF-MS among the samples were consistent (Figure A6B), demonstrating good correspondence. Although medelamine A and tetradecyl-amine had higher scores than *N*,*N*-dimethyldodecylamine when using the genDB, the latter one was deemed more reliable. Moreover, the MS/MS spectrum of *N*,*N*-dimethyldodecylamine shared the same pattern with its homologue *N*,*N*-dimethyltetradecylamine, which was confirmed by a reference standard (Figure A7). Based on this similarity, the peak 270.3173 *m*/*z* at 5.884 min was identified and confirmed as *N*,*N*-dimethylhexadecylamine. Many other matches were found using this method, providing high-confidence identifications by combining library and retention index match in GC–MS and in silico MS/MS spectra.

### Chemicals Associated with Plastic Packaging Database as a Useful Structure Database

To help characterize plastic-related chemicals, the chemicals associated with plastic packaging database (cppDB) compiled by Groh et al.^27^ can be useful. However, the initial version lacks vital structural information, such as Smiles and InChIKey, which is essential for in silico fragmentation. Hence, we added this information to cppDB and reorganized it to be compatible with MS-FINDER, which is available in https://zenodo.org/record/4454648.

**Figure 1:**
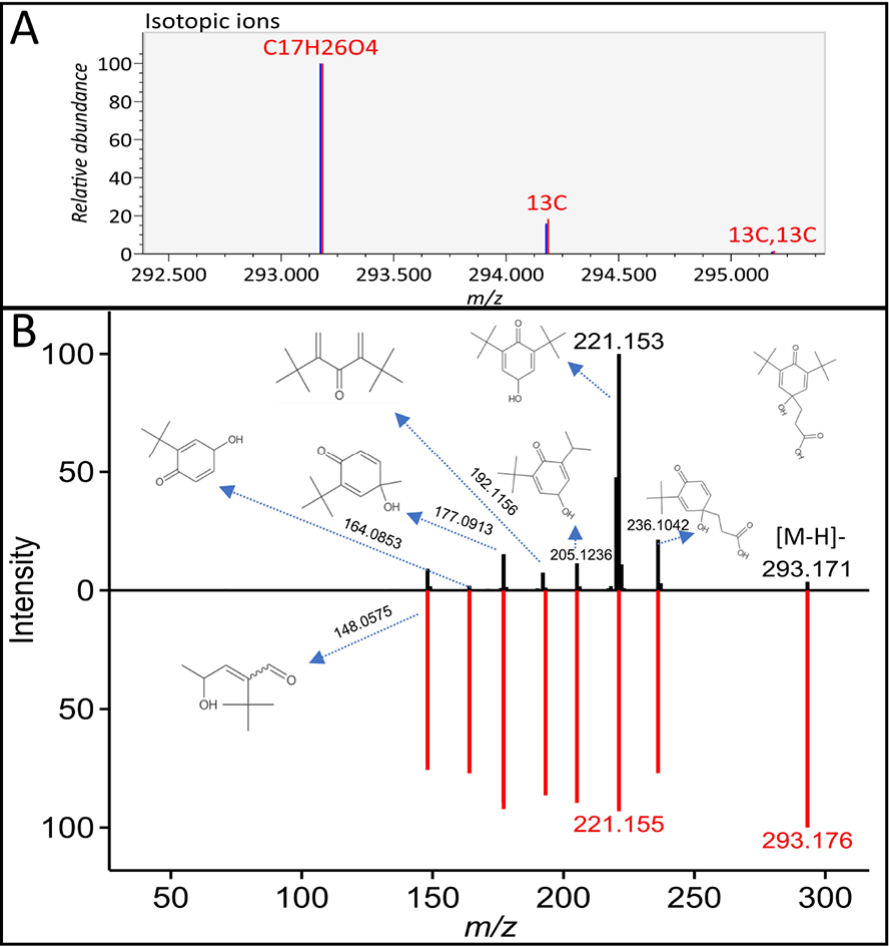
Isotope match (A) and in silico fragmentation (B) of compound **35**.

To showcase the utility of the cppDB, the identification of compound **35** is shown below as an example using the database. The peak 293.1735 *m*/*z* at 6.832 min in negative mode had a formula of C_17_H_26_O_4_ with good isotope match (Figure 1A). While many candidates with scores of >6 were found using the genDB, using the cppDB led to the identification of compound **35** (Figure 1B), which has been reported as a hydrolysate of compound **41**, a degradation product of a common polyolefin antioxidant, Irganox 1010.^31,32^ Compound **41** and Irganox 1010 were also identified in this set of samples. In MS-CleanR, identifications using volDB, cppDB, and genDB were merged, and the unknown was automatically identified as compound **35** with a weight of 1.5 for the cppDB, while the genDB candidates had a weight of 1. The identification was confirmed by comparing the exact mass, retention time, and MS/MS spectrum of the hydrolysis (70 °C for 1 h in water) product of compound **41** to this peak.

Similarly, many other compounds were identified, such as lauric acid diethanolamide. Although some commonly used polymer additives, e.g., Irgafos 168, Irganox 1010, and Chimassorb 81 were identified by library matching, they can also be automatically identified by combining MS-FINDER and the cppDB. It should be noted, however, that the confirmation of identification through a reference standard is the only foolproof method. Nevertheless, in situations where reference standards are not available, combining in silico MS/MS and the cppDB can increase the confidence level of identification in plastic materials.

### Use of Generic Databases

**Figure 2:**
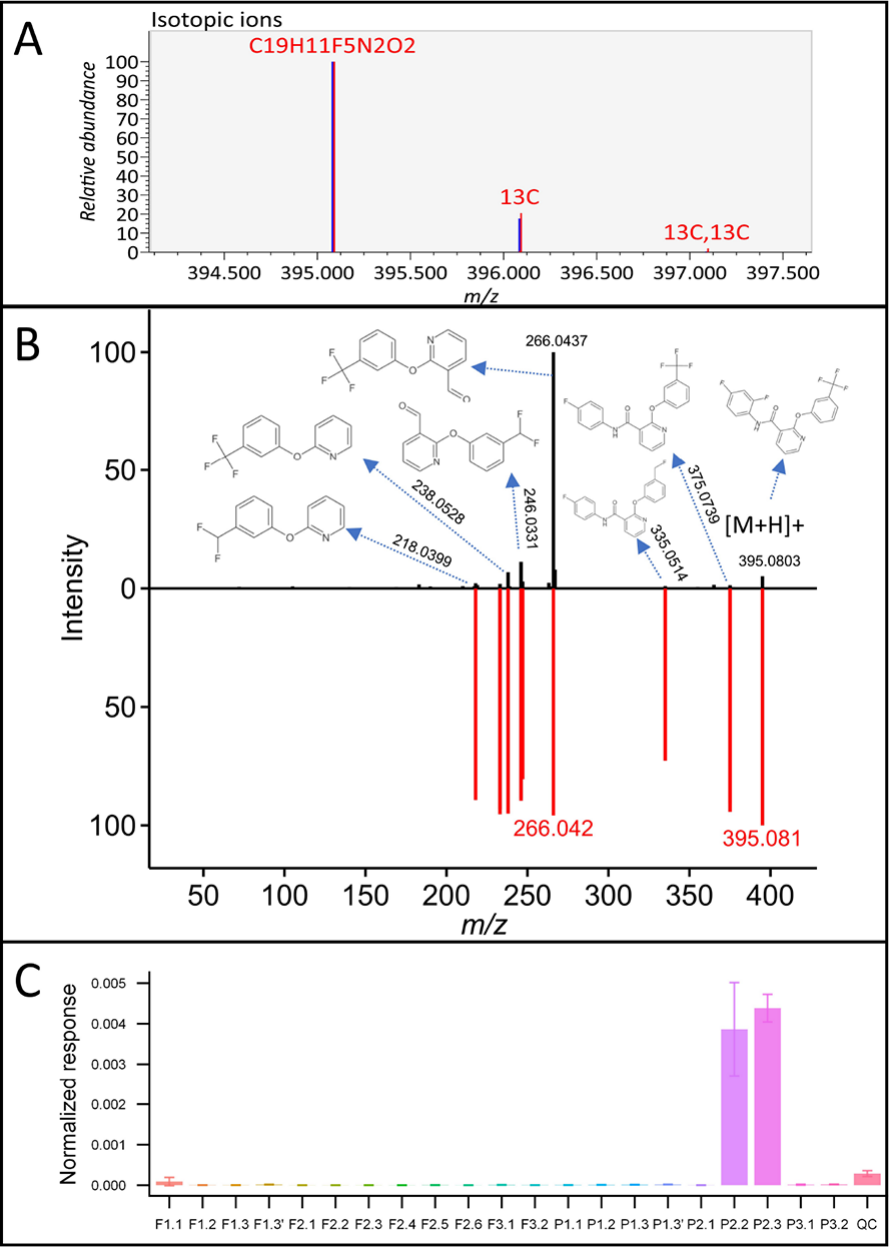
Identification of diflufenican: isotope match (A); in silico fragmentation match by MS-FINDER (B); distribution of this compound across samples (C).

Recycled plastics may contain environmental contaminants and food residues in addition to plastic-related chemicals. Therefore, only generic databases containing information related to food, environment, and natural product were used. Several compounds were identified by this way, and some of them were later confirmed by reference standards. For instance, C_19_H_11_F_5_N_2_O_2_ was found to be the best formula for the peak 395.0808 *m*/*z* at 7.215 min in the positive mode (Figure 2A). Within the genDB, diflufenican was the only candidate that had in silico MS/MS matching the experimental spectrum of the unknown (Figure 2B). Some pesticides/drugs were identified in these samples, especially in P2.2 and P2.3. Therefore, it was not surprising to detect other pesticides. Since this unknown was only found in P2.2 and P2.3 (Figure 2C), diflufenican, an herbicide, was a good candidate for this unknown, and its identification was later confirmed using a certified standard. Similarly, other pesticides/drugs such as pyrifenox and piroxicam were identified in samples P2.2 and P2.3. However, the identification of many other compounds was only based on in silico MS/MS spectra matches (score > 5), and their identification confidences were relatively low. Furthermore, there were 13 compounds that remained unknown, and their spectra are kept in Appendix C, which can be used by MS-FINDER for reproduction and further exploration.

### Pseudo-MRM by MRMPROBS

To determine the presence of an identified substance, it is crucial to examine the samples where it may be present. MS-DIAL provides a means to verify the annotation for each sample (Appendix A, Figure A8). However, some samples may lack annotation due to insufficient intensity or the presence of noise. Assessing such samples based on EIC/TIC can be challenging. In addition, for substances that were not identified through library matches, the MS-DIAL approach cannot be used as there is no annotation data available for each sample. As such, we employed a pseudo-MRM (MRMPROBS) method, which utilized a posterior probabilistic approach to more sensitively, accurately, and automatically determine the presence or absence of a substance in each sample. This approach integrated peak intensity, retention time, precursor–product ion ratio, shape, and coelution similarity to enhance the accuracy of the results.^29^ For instance, the compound 2,2′-methylenebis(6-*tert*-butyl-4-methylphenol) was not identified in samples F2.1 and F2.2 by MS-DIAL (Figure A8), but it was automatically identified in these samples using pseudo-MRM (Figure A9). Moreover, the absence of this compound in sample F2.4 was also confirmed.

In the migration samples, compounds identified in the extracts (by matching libraries) were also subjected to pseudo-MRM analysis, in addition to those characterized in the migrates. While some of these compounds did not have representative spectra for structural elucidation, they were detected in the simulants by the pseudo-MRM. Examples include azoxystrobin and ethyl 4-(dimethylamino)benzoate (Table 1). Essentially, the pseudo-MRM functioned as a sensitive targeted analysis in this context.

### Efficiency of Extra Decontamination on Chemical Removal

Previously, the cleaning efficiency of extra decontamination on volatile compounds was evaluated.^12^ Herein, the non-volatile profiles were assessed. All identified substances and their presence (by pseudo-MRM) in the extracts are listed in Appendix D, with some excluded from Table 1 as they were not detected in the migrates and may not pose a risk to human health. Similarly, *N*-phenyl-2-naphthylamine exhibited significantly higher intensity in both flake and pellet samples following extra decontamination (Figure A10). It was suspected to be a contaminant or reaction product resulting from the extra decontamination process. Another compound that caught our attention was pyriproxyfen, an insecticide, which showed higher intensity in F1.3′ but not in P1.3′ (Figure A11) and was suspected to be an accidental contaminant. Moreover, high-molecular weight substances such as octocrylene and diisodecyl phthalate were not significantly reduced as previously discussed,^12^ which is expected as non-volatile compounds are more difficult to remove. Many pesticides such as sebuthylazine, were only detected in the extracts (Figure A10) but not in the migration (Table 1) likely due to low responses in the extracts. For example, propiconazole showed a strong response in P2.2 and P2.3 (Figure A12), but its presence in other extracts was confirmed by sensitive pseudo-MRM. However, since it was not found in migration, it may pose a risk to humans.

### Quantification of the Migrants

Quantification details including linear range, determined coefficient (*R*^2^), LOD, and LOQ are shown in Appendix E. In line with the previous study,^12^ samples from company 2 contained many high-concern substances (level V and IV) not found in milk bottle-origin rHDPE (Table S1). This can be attributed to the high proportion of non-milk bottle plastics from the agriculture field in these samples. To minimize contamination, better separation of recycled materials is necessary. In company 1 and company 3 samples, the main risks came from two endocrine disruptors, octocrylene and 2-ethylhexyl-4-methoxycinnamate, and they were not significantly reduced by the extra decontamination. For other migrants with SML in EU 10/2011, their migration was all below the SML. Moreover, only few compounds migrated to 3% acetic acid, and their migrations were quite low and might not be risky for human health.

## Conclusions

Aided by our in house MS/MS library (publicly available now), the developed R package *mspcompiler*, and our data analysis workflow, non-volatile compounds in rHDPE were characterized for the first time using non-targeted screening. Manual examination of the spectra is necessary during feature cleaning when dealing with data independent acquisition data. The list of identified compounds in the same samples using GC–MS was employed as a structure database for the in silico fragmentation tool (MS-FINDER) and automatically found correspondences for some unknowns in LC-QTOF-MS, helping us avoid spending plenty of time on elucidating compounds that were already known in the samples. The cppDB was valuable for identifying plastic-related compounds in the recycled plastics and is expected to be helpful for the investigations of other plastic packaging. However, this strategy only works for known unknowns, not unknown unknowns, which is much more challenging as they might not be present in any existing structure databases. Once identified, pseudo-MRM using precursor–product ion pairs was more sensitive and precise for determining the presence of compounds in the samples. Many high-risk substances were detected in samples with high amounts of non-milk-bottle plastics, demonstrating the significance of good sorting. Even so, high-molecular weight substances such as octocrylene are still obstacles for the food contact applications of rHDPE as they are not easy to remove.

## Supporting Information Available

Experimental details, supplementary figures, and supplementary tables (PDF)
Extended version of Table 1, showing more details such as mass error between the acquired and theoretical mass of the precursor ions, CID (ID in Pubchem), SMILES, and InChIKey of the identified compounds; spectra of the unknowns; chemicals identified in the extracts; quantification details including linear range, determined coefficient (*R*^2^), LOD, and LOQ (ZIP)
