# Supplementary material for: Combination of Structure Databases, In Silico Fragmentation, and MS/MS Libraries for Untargeted Screening of Non-Volatile Migrants from Recycled High-Density Polyethylene Milk Bottles
Source: Anal Chem. 2023 Jun 1;95(23):8780–8. doi: 10.1021/acs.analchem.2c05389 (PMC10267890; doi:10.1021/acs.analchem.2c05389)
Supplement: Supplementary file 1 — ac2c05389_si_001.pdf [file ac2c05389_si_001.pdf]

Supporting Information

Combination of Structure Databases, In-Silico Fragmentation, and MS/MS Libraries for Untargeted Screening of Non-volatile Migrants from Recycled High-Density Polyethylene Milk bottles

Qi-Zhi Su†,††, Paula Vera†, Cristina Nerín†*

† Department of Analytical Chemistry, GUIA Group, I3A, EINA, University of Zaragoza, María de Luna 3, 50018, Zaragoza, Spain

†† National Reference Laboratory for Food Contact Material (Guangdong), Guangzhou Customs Technology Center, Guangzhou 510075, China

There are 5 supplementary materials for this article:

1. **Appendix A (this document):** contains more experimental details, supplementary figures, and supplementary tables
2. **Appendix B:** an extended version of Table 1, showing more details such as mass error between the acquired and theoretical mass of the precursor ions, CID (ID in Pubchem), SMILES, and InChIKey of the identified compounds
3. **Appendix C:** spectra of the unknowns, which can be read by MS-FINDER for further exploration
4. **Appendix D:** chemicals identified in the extracts
5. **Appendix E:** quantification details including linear range, determine coefficient (R2), LOD, and LOQ

Appendix A

1. Reagents used

The following analytical standards were purchased from Sigma-Aldrich (Madrid, Spain): aminophenazone (58-15-1), o-anisidine (90-04-0), 2,4-dimethylbenzenamine (95-68-1), caprolactam (105-60-2), caffeine (58-08-2), N,N-bis (2-hydroxy- ethyl)dodecylamine (1541-67-9), dimethyldibenzyli- dene sorbitol (135861-56-2), N-[3-(dimethylamino) propyl]dodecanamide (3179-80-4), N,N-dimethylte- tradecylamine (112-75-4), pyrimethanil (53112-28-0), N,N-dimethylhexadecylamine (112-69-6), N-me-thyldidecylamine (7396-58-9), 3,3'-dichlorobenzidi-ne (91-94-1), tebuconazole (80443-41-0), 7,9-di-tert-butyl-1-oxaspiro(4,5)deca-6,9-diene-2,8-dione (82304-66-3), lauric acid diethanolamide (120-40-1), diflufenican (83164-33-4), tributyl citrate (77-94-1), tributyl acetylcitrate (77-90-7), octocrylene (6197-30-4), avobenzone (70356-09-1), 2-stearoyl-glycerol (621-61-4), Chimassorb 81 (1843-05-6), dioctyl phthalate (117-84-0), bis(2-ethylhexyl) adi-pate (103-23-1), erucamide (112-84-5), Irgafos 168 (31570-04-4), Irganox 1010 (6683-19-8), tris(2,4-ditert-butylphenyl)phosphate (95906-11-9), Irganox 1076 (2082-79-3), glycerol dihexanoate (502-52-3), ethyl 4-(dimethylamino)benzoate (10287-53-3), oxybenzone (131-57-7), 2-ethylhexyl-4-methoxycin-namate (83834-59-7), palmitamide (629-54-9), palmitic acid (57-10-3), propanil (709-98-8), oleic acid (112-80-1), thiabendazole (148-79-8), 1-octyl-pyrrolidin-2-one (2687-94-7), docosanamide (3061-75-4), diisodecyl phthalate (89-16-7), 1,2,3-trideoxy-4,6:5,7-bis-o-[(4-propylphenyl)methylene]-nonitol (NX 8000 K, 882073-43-0), and 2,5-bis(5-tert-butyl-benzoxazol-2-yl)thiophene (7128-64-5). 3-(3,5-di-tert-butyl-4-hydroxyphenyl)propionic acid (20170-32-5) was from Enamine (Riga, Latvia).

2. The *mspcompiler* R package

As detailed in Github (https://github.com/QizhiSu/mspcompiler), the objective of the *mspcompiler* R package is to offer means to compile either EI or tandem mass spectral libraries from various sources, such as NIST (if you have it installed), MoNA, and GPNS, and organize them into a neat and up-to-date *.msp file that can be used in MS-DIAL.

If you have NIST library installed, you can follow the step-by-step instruction described in the abovementioned website to convert the NIST library into *.msp format. However, the converted version does not contain SMILES (used for visualising the chemical structure in MS-DIAL) and retention index (RI, useful for improving the reliability of the identification in GC-MS data processing). For this reason, the package offers a function to extract chemical structures and assign the SMILES to the *.msp file accordingly, such that the analyst can have a general idea about the chemical structure of the candidates, which is useful for data interpretation. Different manipulations may be required depending on the specific publicly available EI libraries that need to be compiled. For instance, if you are working with the MoNA library, you may need to use the reorganize_mona() function to reorganize the SMILES and clean the chemical name.

For EI library, the package offers a way to extract reference RI in the NIST and assign them to the *.msp file accordingly based on the polarity of the column used. For those chemicals that do not have reference RI in NIST, then you can export their SMILES and estimate their RI using prediction models, for example the one developed by (Matyushin, Dmitriy D., Anastasia Yu Sholokhova, and Aleksey K. Buryak. 2019. "A Deep Convolutional Neural Network for the Estimation of Gas Chromatographic Retention Indices." Journal of Chromatography A 1607: 460395. https://doi.org/10.1016/j.chroma.2019.460395.), and then assign the predicted RI to the *.msp file

accordingly. In practice, rounding the reference RI to the nearest integer while keeping two decimal places for the predicted RI can be helpful when manually checking the results. This allows for differentiation between the reference and predicted values in MS-DIAL.

For MS/MS library, after converting the NIST library into the *.msp format, you may need to separate the library into 2 parts based on the polarity since MS-DIAL processes positive and negative mode data separately. If you are working with the GNPS library, in addition to the polarity separation, you may need to convert it from *.mgf to *.msp format.

Once each library is cleaned and reorganized, you can combine and export them as *.msp format accordingly.

3. Constructing of in-house library

The in-house library was built following the strategy proposed by (Tada, Ipputa et al. 2019. "Creating a Reliable Mass Spectral–Retention Time Library for All Ion Fragmentation-Based Metabolomics." Metabolites 9(251): 1–15.). The standards were, in principle, prepared in methanol, with 10 standards being mixed together. In a mixture, no repeated molecular formula is allowed to avoid any confusion. These mixtures with 5 different concentrations were then injected into the UPLC-QTOF-MS system using the following conditions, which are identical to the ones used in this study:

A Waters Acquity UPLC equipped with an AtlantisTM premier BEH C18 AX column (2.1 × 100 mm) of 1.7 µm particle size (Milford, MA, USA) was employed for the separation. Column temperature was set at 40 °C under the flow of 0.3 mL/min. Water and methanol, both spiked with 0.1 % formic acid, were the mobile phase A and B, respectively, for both positive and negative modes. A 13 min run was used with the following gradient elution: initial mobile phase A/B 95/5 was shifted to A/B 100/0 in 7 min, kept for 4 min, then dropped to the initial mobile phase in 0.1 min, and maintained for additional 1.9 min to get the system ready for the next injection. Injection volume was 10 µL.

The QTOF-MS was coupled to the UPLC by an electro spray ionization (ESI) probe. The conditions employed were as follows: resolution mode, capillary voltage 3.0 kV, sampling cone voltage 45 V, extraction cone 4.0 V, source temperature 150 V, desolvation temperature 350 °C, cone gas flow rate of 40 L/h, and the desolvation gas flow rate of 600 L/h. Data independent analysis (DIA, i.e., MSE), with low energy at 6 V and ramp high energy at 10-30V , was used for data acquisition and masses from 50 to 1200 Da were scanned. Leucine enkephalin (CAS 58822-25-6) at 2 ng/mL was employed for on-line mass correction. Test-mix from Waters was injected every 20 injections to ensure accuracy of the data.

The data was processed by MS-DIAL using post identification mode, where a *.txt file specifying the name, exact mass of the precursor ions, adduct, InChIKey, Formula, and SMILES of the standards is required. Adducts considered in negative mode were [M-H]-, [M+FA-H]-, [M+Hac-H]-, [2M-H]-, [2M+FA-H]-, and [2M+Hac-H]-, while in positive mode were [M+H]+, [M+NH4]+, [M+Na]+,

Metabolite	MZ	RT	Adduct	InChIKey	Formula	SMILES
3-Carbamoylbenzoic acid	164.035317	-1	[M-H]-	FVUKYCZRWSQGAS-UHFFFAOYSA-N	C8H7NO3	C1=CC(=CC(=C1)C(=O)O)C(=O)N
Cyasorb UV 1084	570.292117	-1	[M-H]-	DPLLDVMBMPQDCO-UHFFFAOYSA-L	C32H51NNiO2S	CCCCN.CC(C)(C)CC(C)(C)C1=CC(=C(C=C1)[O-])SC2=C(C=CC(=C2)C(C)(C)CC(C)(C)C)[O-].[Ni+2]
Tinuvin P	224.082936	-1	[M-H]-	MCPKSFINULVDNX-UHFFFAOYSA-N	C13H11N3O	CC1=CC(=C(C=C1)O)N2N=C3C=CC=CC3=N2
Antioxidante L	513.398306	-1	[M-H]-	GHKOFFNLGXMVNJ-UHFFFAOYSA-N	C30H58O4S	CCCCCCCCCCCCOC(=O)CCSCCC(=O)OCCCCCCCCCCCC
bis(2-ethylhexyl) adipate	369.301034	-1	[M-H]-	SAOKZLXYCUGLFA-UHFFFAOYSA-N	C22H42O4	CCCCC(CC)COC(=O)CCCCC(=O)OCC(CC)CCCC
Dimethyl adipate	173.081933	-1	[M-H]-	UDSFAEKRVUSQDD-UHFFFAOYSA-N	C8H14O4	COC(=O)CCCCC(=O)OC
2,2,4-Trimethyl-1,3-pentanediol diisobutyrate	285.207133	-1	[M-H]-	OMVSWZDEEGIJJI-UHFFFAOYSA-N	C16H30O4	CC(C)C(C(C)(C)COC(=O)C(C)C)OC(=O)C(C)C
bis (2ethylhexyl) sebacate	425.363634	-1	[M-H]-	VJHINFRRDQUWOJ-UHFFFAOYSA-N	C26H50O4	CCCCC(CC)COC(=O)CCCCCCCCC(=O)OCC(CC)CCCC
dibutyl phtalate	277.144533	-1	[M-H]-	DOIRQSBPFJWKBE-UHFFFAOYSA-N	C16H22O4	CCCCOC(=O)C1=CC=CC=C1C(=O)OCCCC
NX8000	483.275213	-1	[M-H]-	PIYNPBVOTLQBTC-UHFFFAOYSA-N	C29H40O6	CCCC1C2C(C(OC(O2)C3=CC=C(C=C3)CCC)C(CO)O)OC(O1)C4=CC=C(C=C4)CCC
3-Carbamoylbenzoic acid	210.040794	-1	[M+FA-H]-	FVUKYCZRWSQGAS-UHFFFAOYSA-N	C8H7NO3	C1=CC(=CC(=C1)C(=O)O)C(=O)N
Cyasorb UV 1084	616.297594	-1	[M+FA-H]-	DPLLDVMBMPQDCO-UHFFFAOYSA-L	C32H51NNiO2S	CCCCN.CC(C)(C)CC(C)(C)C1=CC(=C(C=C1)[O-])SC2=C(C=CC(=C2)C(C)(C)CC(C)(C)C)[O-].[Ni+2]
Tinuvin P	270.088413	-1	[M+FA-H]-	MCPKSFINULVDNX-UHFFFAOYSA-N	C13H11N3O	CC1=CC(=C(C=C1)O)N2N=C3C=CC=CC3=N2
Antioxidante L	559.403783	-1	[M+FA-H]-	GHKOFFNLGXMVNJ-UHFFFAOYSA-N	C30H58O4S	CCCCCCCCCCCCOC(=O)CCSCCC(=O)OCCCCCCCCCCCC
bis(2-ethylhexyl) adipate	415.306511	-1	[M+FA-H]-	SAOKZLXYCUGLFA-UHFFFAOYSA-N	C22H42O4	CCCCC(CC)COC(=O)CCCCC(=O)OCC(CC)CCCC
Dimethyl adipate	219.08741	-1	[M+FA-H]-	UDSFAEKRVUSQDD-UHFFFAOYSA-N	C8H14O4	COC(=O)CCCCC(=O)OC
2,2,4-Trimethyl-1,3-pentanediol diisobutyrate	331.21261	-1	[M+FA-H]-	OMVSWZDEEGIJJI-UHFFFAOYSA-N	C16H30O4	CC(C)C(C(C)(C)COC(=O)C(C)C)OC(=O)C(C)C
bis (2ethylhexyl) sebacate	471.369111	-1	[M+FA-H]-	VJHINFRRDQUWOJ-UHFFFAOYSA-N	C26H50O4	CCCCC(CC)COC(=O)CCCCCCCCC(=O)OCC(CC)CCCC
dibutyl phtalate	323.15001	-1	[M+FA-H]-	DOIRQSBPFJWKBE-UHFFFAOYSA-N	C16H22O4	CCCCOC(=O)C1=CC=CC=C1C(=O)OCCCC
NX8000	529.28069	-1	[M+FA-H]-	PIYNPBVOTLQBTC-UHFFFAOYSA-N	C29H40O6	CCCC1C2C(C(OC(O2)C3=CC=C(C=C3)CCC)C(CO)O)OC(O1)C4=CC=C(C=C4)CCC

[M+K]+, [2M+H]+, [2M+NH4]+, [2M+Na]+, and [2M+K]+. The InChIKey, Formula, and SMILES were retrieved from Pubchem using the webchem R package.

Then, the data was manually checked considering the following criteria:

1). If the Mass error is within the tolerance (5 ppm)

2). If the intensity increases with higher concentration

3). If the main fragment ions are explainable by MS-FINDER's in-silico fragmentation.

When all these criteria were met, the post identification was deemed confident, and the peak along with its Name, Adduct, InChIKey, SMILES, Formula, and MS/MS spectra were exported to MS-FINDER. Sometimes, MS-DIAL allocated a wrong adduct, then we would modify it in MS-FINDER, and saved them as a *.msp file. Finally, the MS-LIMA software was employed to check if there is any error in the library and to convert the mass of the precursor ions into the theoretical one.

4. Compilation of MS/MS libraries used for identification in MS-DIAL

Following the mspcompiler R package manual (https://github.com/QizhiSu/mspcompiler), we consolidated and restructured MS/MS libraries from various sources, including those compiled by the MS-DIAL developer, downloaded from GNPS (https://gnps.ucsd.edu/ProteoSAFe/libraries.jsp), and from NIST 17. We also incorporated an in-house library containing 449 and 172 chemicals associated with food packaging in positive and negative modes, respectively. The compiled libraries, both positive and negative, were employed for the identification accordingly.

5. Converting GC-MS identified substances into a structure database used by MS-FINDER

In order to correlate LC-MS signals to a list of volatile and semi-volatile compounds for a given sample, a common approach is to compute the molecular formula from the LC-MS signals and manually search for the corresponding formula in the list of compounds. However, this approach may not be sufficient, as relying solely on the molecular formula may not accurately identify the correct candidate. While one could download the *.mol file of the candidate and use an in-silico fragmentation tool to confirm its viability, this method can be time-consuming and manual.

To address this issue, we propose a new method using MS-FINDER, which utilizes the list of volatile and semi-volatile compounds as a structure database. We have developed a R function, namely export4msfinder in the labtools R package (https://github.com/QizhiSu/labtools.) to convert any list of chemicals into a structure database to be used in MS-FINDER (this function can also be used for converting other list of chemicals that are related to the samples under investigation). With this method, the structure information of the compounds can be automatically retrieved, and after computing the molecular formulas, MS-FINDER will use this database to computationally fragment structures with the same molecular formula and rank them based on factors such as in-silico fragmentation probability. This approach can significantly reduce the manual interpretation required and increase the efficiency and accuracy of the compound identification process.

6. Supplementary figures

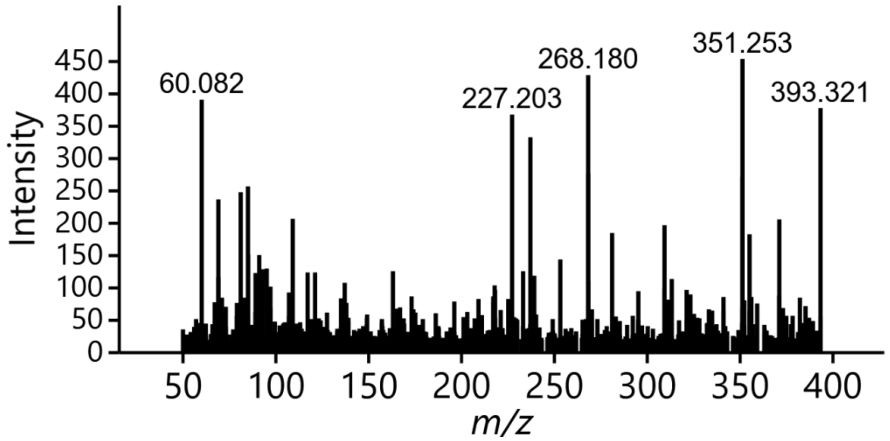

Figure S1. Example of noisy MS/MS spectrum

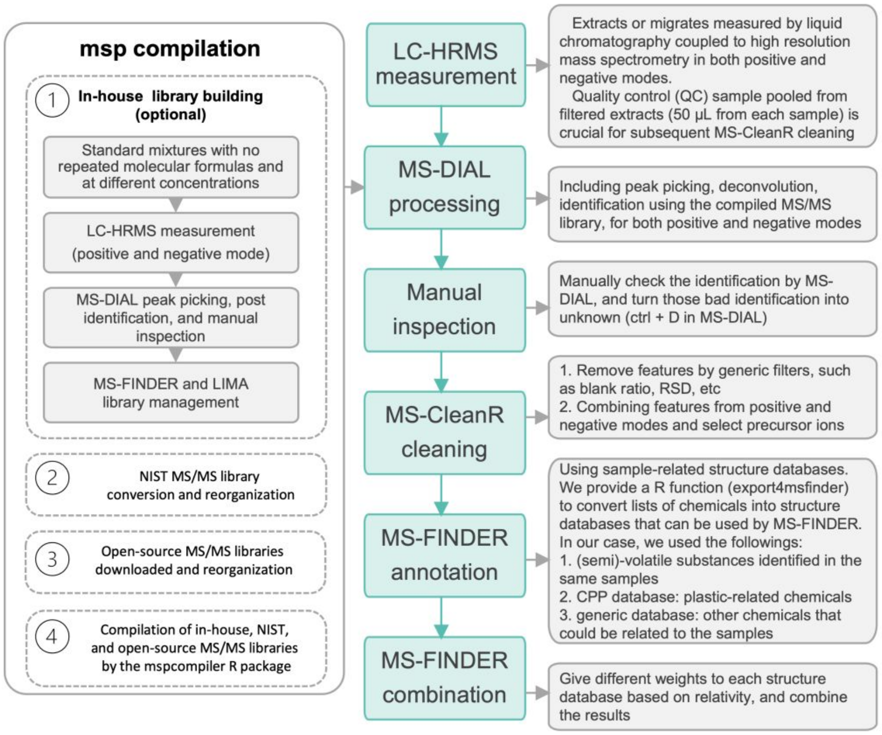

Figure S2. Flow chart for the identification process

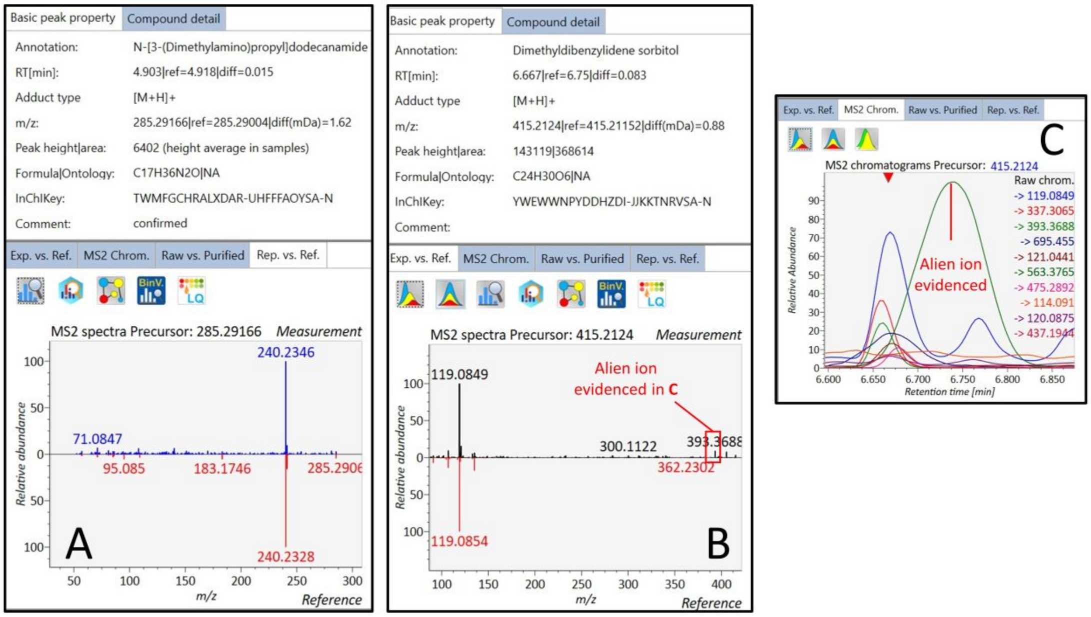

Figure S3. Negative effect of tiny and noisy signals on matching scores in MS-DIAL: example of N-[3-(dimethylamino)propyl]dodecanamide (A); example of dimethyldibenzylidene sorbitol (B); evidence of alien ions (393.3688) in the MS/MS spectra of dimethyldibenzylidene sorbitol (C)

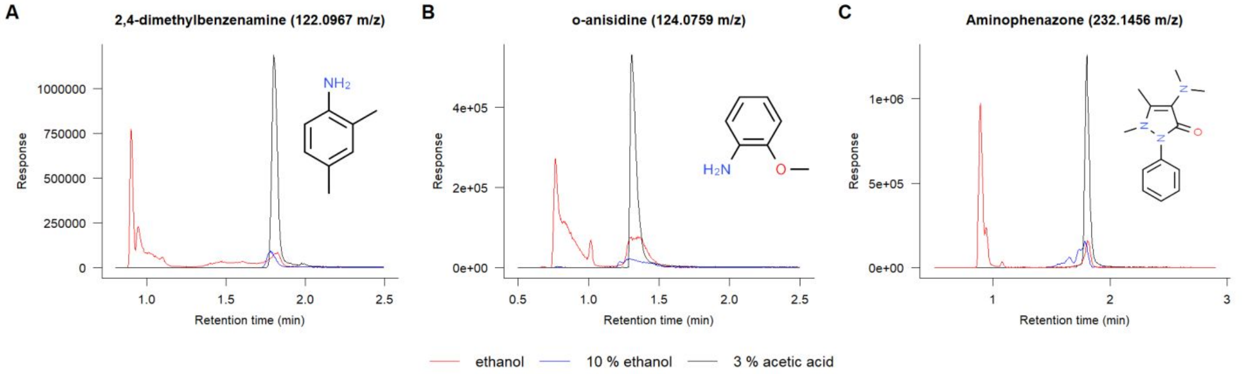

Figure S4. Chromatograms of 2,4-dimethylbenzenamine (A), o-anisidine (B), and aminophenazone (C) in 3 solvents. The 10 % ethanol standards were obtained by directly diluting the ethanol standards 10 times.

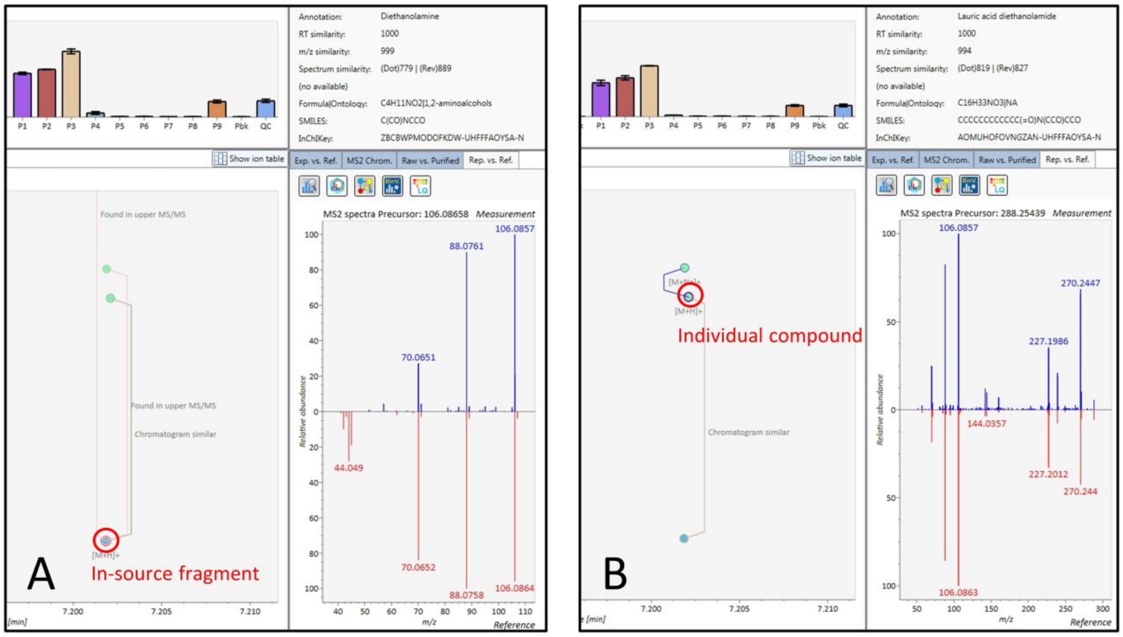

Figure S5. Library matching results: mis-identification of an in-source fragment as a single compound (A); identification of a single compound (B)

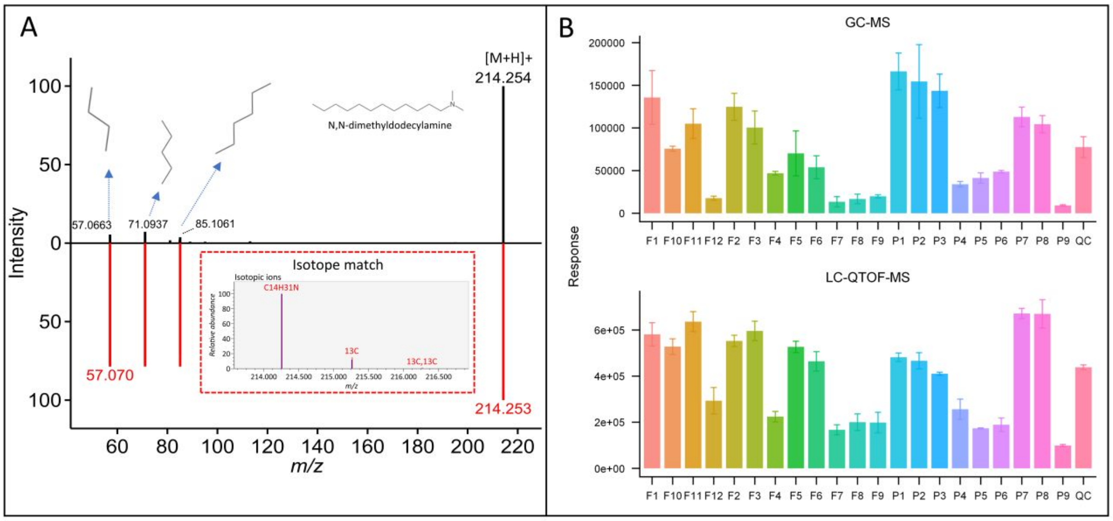

Figure S6. Identification of N,N-dimethyldodecylamine: In-silico fragmentation match by MS-FINDER (A) and the distribution of this compound among samples in GC-MS and LC-QTOF-MS (B)

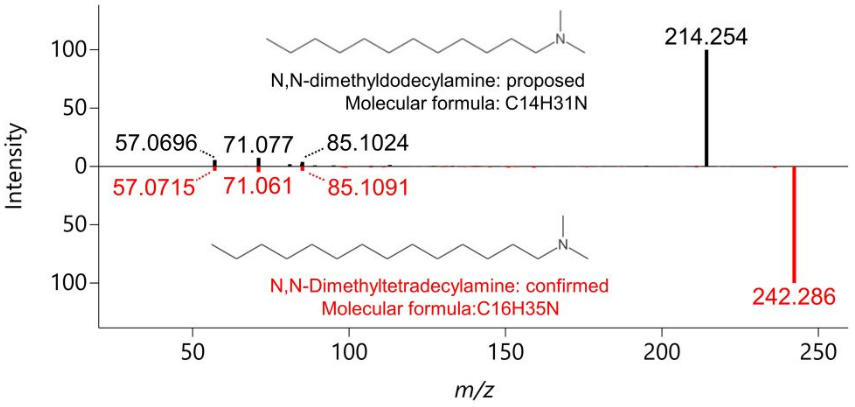

Figure S7. MS/MS spectra of the two homologs N,N-dimethyltetradecylamine (confirmed by standard) and N,N-dimethyldodecylamine

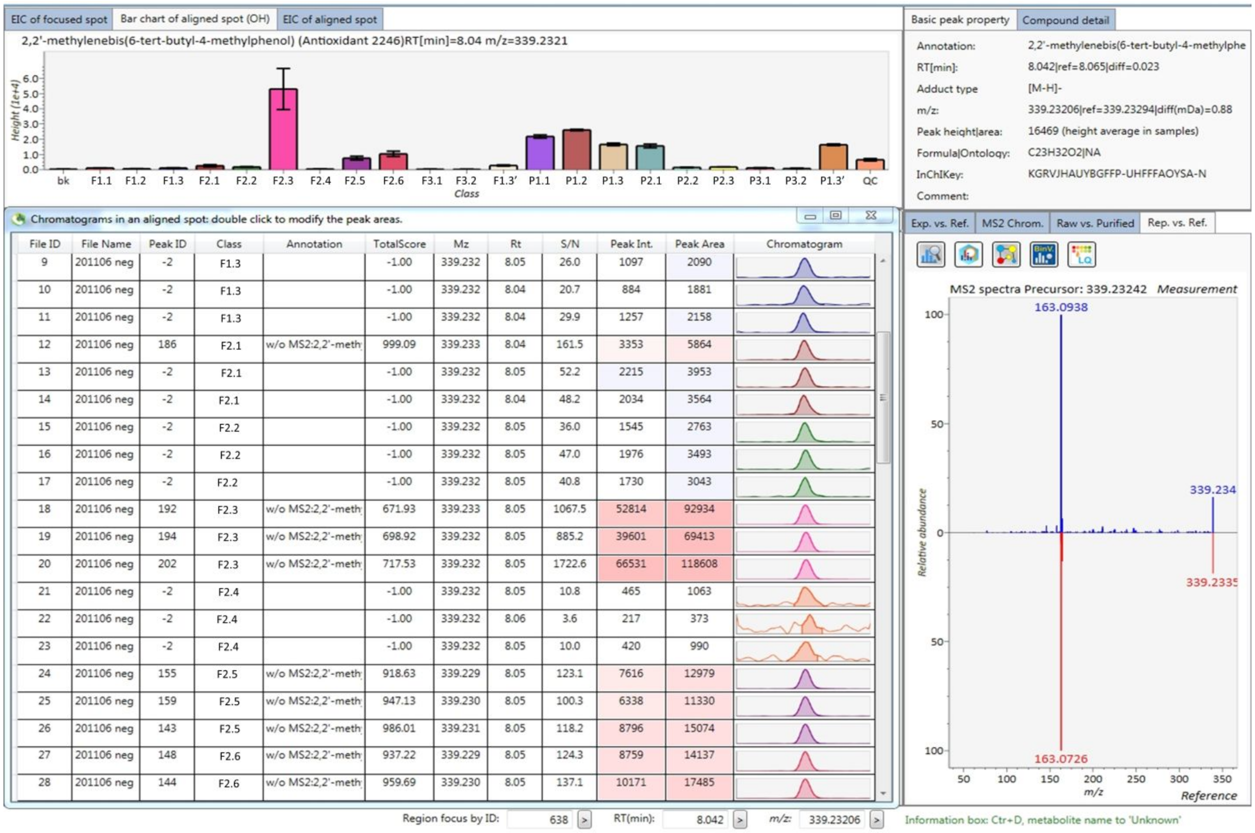

Figure S8. Evaluating the presence/absence of 2,2'-methylenebis(6-tert-butyl-4-methylphenol) (Antioxidant 2246) in each sample by MS-DIAL

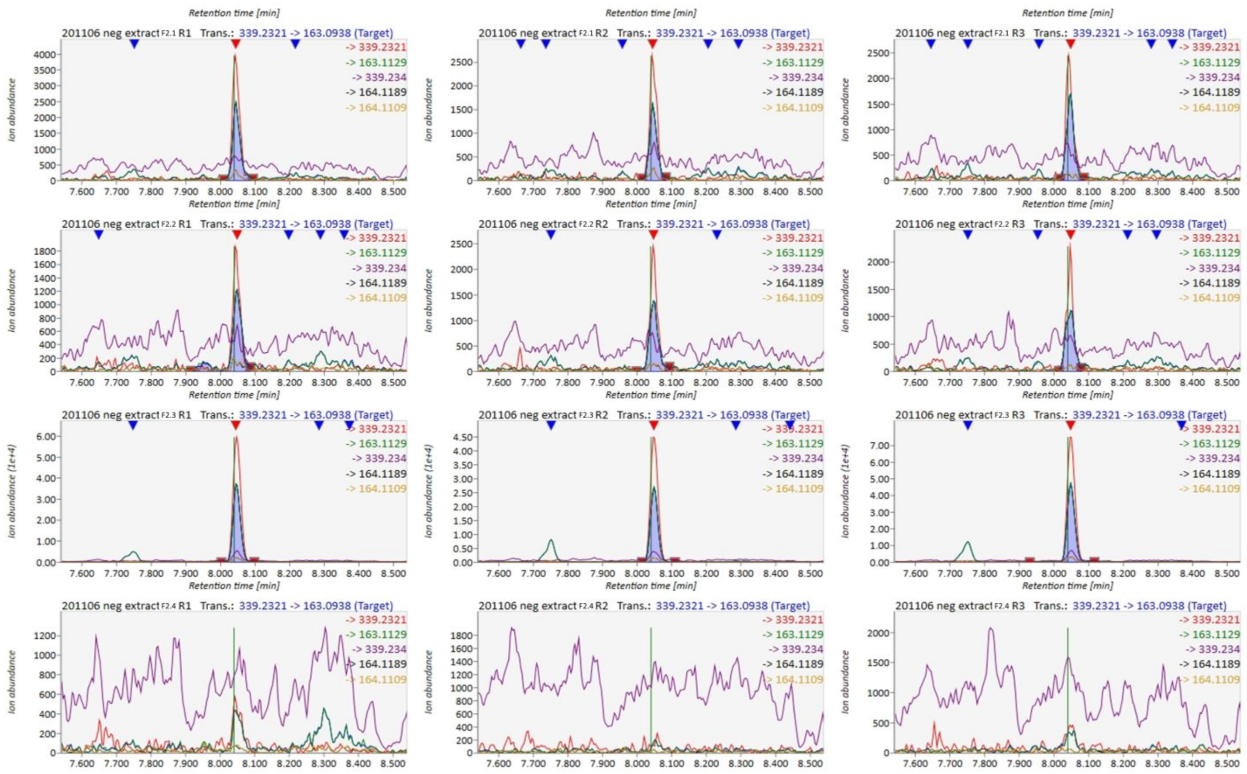

Figure S9. Evaluating the presence/absence of 2,2'-methylenebis(6-tert-butyl-4-methylphenol) (Antioxidant 2246) in each sample by MRMPROBS (pseudo-MRM)

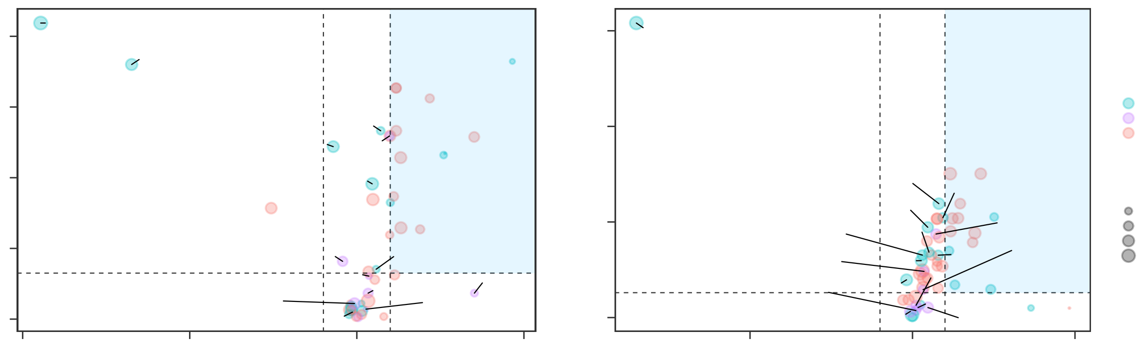

Note: Fold change is expressed as no extra decontamination versus extra decontamination; the size of the circles is mapped to the average peak area of the samples that applied extra decontamination

Figure S10. Efficiency of extra decontamination: fold change analysis by volcano plot on flakes (A); fold change analysis by volcano plot on pellets (B)

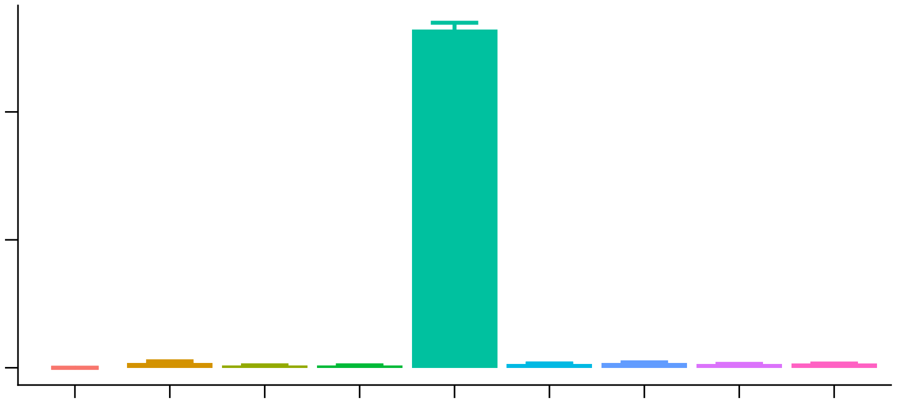

Figure S11. Bar chart of pyriproxyfen across company1 samples

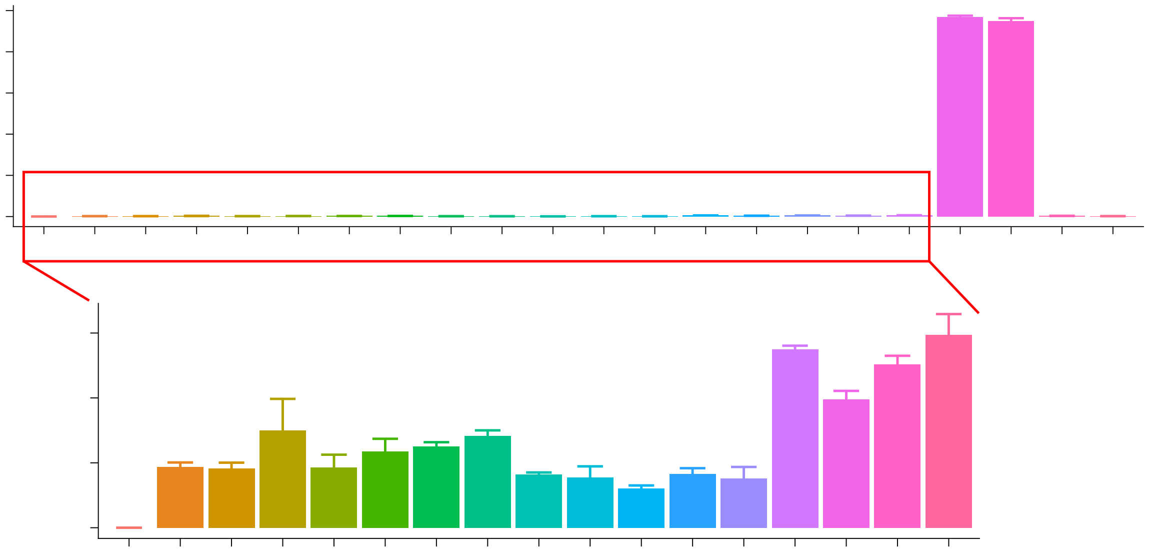

Figure S12. Bar chart of propiconazole across samples (extracts)

7. Supplementary tables

Table S1. Quantification of the migrants in 95 % and 3% acetic acid

Name	Company 1								Company 2						Company 3				Remarks
	EP1.1	EP1.2	EP1.3	EP1.3'	HP1.1	HP1.2	HP1.3	HP1.3'	EP2.1	EP2.2	EP2.3	HP2.1	HP2.2	HP2.3	EP 3.1	EP3.2	HP3.1	HP3.2	
**Aminophenazone**	n.d.	n.d.	n.d.	n.d.	n.d.	n.d.	n.d.	n.d.	n.d.	89 ± 3	98.2 ± 4.7	n.d.	n.d.	n.d.	n.d.	n.d.	n.d.	n.d.	IV
**o-Anisidine**	n.d.	n.d.	n.d.	n.d.	n.d.	n.d.	n.d.	n.d.	41.6 ± 3.4	10.6 ± 5.2	7 ± 2.1	47.2 ± 3.1	25.8 ± 3.7	27 ± 4.3	n.d.	n.d.	n.d.	n.d.	V
2,4-Dimethylbenzenamine	1.6 ± 0.04	1.5 ± 0.02	< LOQ	n.d.	1.3 ± 0.1	1 ± 0.2	0.6 ± 0.3	< LOQ	71.6 ± 0.7	21.8 ± 0.8	19 ± 0.2	89.5 ± 4.8	39.5 ± 0.4	38.2 ± 0.8	< LOQ	< LOQ	< LOQ	< LOQ	II
**Thiabendazole**	n.d.	n.d.	n.d.	n.d.	n.d.	n.d.	n.d.	n.d.	n.d.	n.d.	n.d.	n.d.	3.4 ± 0.4	3.3 ± 0.1	n.d.	n.d.	n.d.	n.d.	IV
**Caprolactam**	n.d.	n.d.	n.d.	n.d.	n.d.	n.d.	n.d.	n.d.	280 ± 24	957 ± 33	933 ± 12	320 ± 0.4	943 ± 8	940 ± 14	n.d.	n.d.	10.6 ± 1.5	9 ± 10.5	II; SML 15
**Caffeine**	n.d.	n.d.	n.d.	n.d.	3.1 ± 0.1	3 ± 0.5	2.8 ± 0.6	n.d.	n.d.	n.d.	n.d.	n.d.	5.5 ± 0.3	5.1 ± 0.1	n.d.	n.d.	n.d.	n.d.	IV
N,N-bis (2-hydroxyethyl)dodecylamine	1.8 ± 0.01	1.2 ± 0.04	1.1 ± 0.01	0.2 ± 0.1	< LOQ	< LOQ	< LOQ	< LOQ	12.2 ± 0.4	1.7 ± 0.3	1.3 ± 0.2	4.1 ± 0.3	< LOQ	< LOQ	n.d.	n.d.	n.d.	n.d.	II
N-[3-(Dimethylamino)propyl]dodecanamide	2.5 ± 0.2	2.2 ± 0.3	2 ± 0.2	1.2 ± 0.1	0.3 ± 0.2	0.2 ± 0.05	0.3 ± 0.01	< LOQ	< LOQ	4.1 ± 0.5	3.9 ± 0.1	0.1 ± 0.01	1.3 ± 0.1	1.4 ± 0.1	n.d.	n.d.	n.d.	n.d.	IV
N,N-Dimethyltetradecylamine	8.9 ± 0.04	9.1 ± 0.5	8.2 ± 0.3	2.5 ± 0.04	6.1 ± 0.2	5.2 ± 0.2	5 ± 0.3	1.8 ± 0.1	2.3 ± 0.3	3.2 ± 0.3	3.1 ± 0.4	2.9 ± 0.2	3.1 ± 0.2	3.1 ± 0.1	10 ± 0.4	11.8 ± 1.8	4.6 ± 0.4	4.8 ± 0.8	II
Pyrimethanil	n.d.	n.d.	n.d.	n.d.	n.d.	n.d.	n.d.	n.d.	n.d.	15.7 ± 1.3	13.4 ± 2.5	n.d.	12.4 ± 2.1	12.3 ± 0.4	n.d.	n.d.	< LOQ	< LOQ	IV
N,N-Dimethylhexadecylamine	10.3 ± 0.3	12.7 ± 1.8	10.1 ± 0.1	6.4 ± 0.4	29 ± 1.3	29.6 ± 0.1	n.d.	n.d.	3 ± 0.2	6.5 ± 0.4	6.6 ± 0.4	n.d.	n.d.	n.d.	2.8 ± 0.1	3.1 ± 0.2	n.d.	n.d.	II
N-methyldidecylamine	10.7 ± 0.2	9.1 ± 0.01	9.5 ± 0.5	8.3 ± 0.05	n.d.	n.d.	n.d.	n.d.	7.3 ± 0.6	3 ± 0.6	2.8 ± 0.1	n.d.	n.d.	n.d.	0.2 ± 0.1	0.2 ± 0.01	n.d.	n.d.	II
**3,3'-dichlorobenzidine**	n.d.	n.d.	n.d.	n.d.	n.d.	n.d.	n.d.	n.d.	209 ± 7	74.9 ± 0.8	73.6 ± 0.1	46.5 ± 0.7	16.8 ± 0.7	17.4 ± 2	n.d.	n.d.	n.d.	n.d.	V
Propanil	n.d.	n.d.	n.d.	n.d.	n.d.	n.d.	n.d.	n.d.	n.d.	861 ± 46	739 ± 44	n.d.	519 ± 9	518 ± 31	n.d.	n.d.	n.d.	n.d.	IV
**Ethyl 4-(dimethylamino)benzoate**	1 ± 0.1	1 ± 0.1	1 ± 0.2	n.d.	n.d.	n.d.	n.d.	n.d.	1.5 ± 0.1	1.6 ± 0.2	1.4 ± 0.1	n.d.	n.d.	n.d.	n.d.	n.d.	n.d.	n.d.	II
**Dimethyldibenzylidene sorbitol**	13.4 ± 0.3	13.8 ± 0.8	13.3 ± 0.8	n.d.	n.d.	n.d.	n.d.	n.d.	n.d.	12.5 ± 0.1	12 ± 1.3	n.d.	n.d.	n.d.	n.d.	n.d.	n.d.	n.d.	I; SML 60
**Oxybenzone**	n.d.	n.d.	n.d.	n.d.	n.d.	n.d.	n.d.	n.d.	17.7 ± 1.8	21.4 ± 0.1	19.6 ± 1.6	n.d.	n.d.	n.d.	11.6 ± 1.3	12.4 ± 0.1	n.d.	n.d.	V; SML 6
**1-octylpyrrolidin-2-one**	n.d.	n.d.	n.d.	n.d.	n.d.	n.d.	n.d.	n.d.	n.d.	42.4 ± 3.4	41.5 ± 2.1	n.d.	52.3 ± 2.2	52.3 ± 1	n.d.	n.d.	n.d.	n.d.	IV
Tebuconazole	n.d.	n.d.	n.d.	n.d.	n.d.	n.d.	n.d.	n.d.	n.d.	12.2 ± 0.6	12.4 ± 0.9	n.d.	12.5 ± 0.3	12.5 ± 0.1	n.d.	n.d.	n.d.	n.d.	V
Lauric acid diethanolamide	31.2 ± 2.8	34 ± 2.1	40.2 ± 1.9	4.9 ± 0.4	0.9 ± 0.1	1.2 ± 0.4	1.1 ± 0.03	< LOQ	n.d.	n.d.	n.d.	n.d.	n.d.	n.d.	n.d.	n.d.	n.d.	n.d.	II; SML 5
Diflufenican	n.d.	n.d.	n.d.	n.d.	n.d.	n.d.	n.d.	n.d.	n.d.	38 ± 10.9	42.2 ± 3.1	n.d.	n.d.	n.d.	n.d.	n.d.	n.d.	n.d.	IV
**tributyl citrate**	2.2 ± 0.6	2.9 ± 0.4	2.3 ± 0.2	1.2 ± 0.3	n.d.	n.d.	n.d.	n.d.	4.5 ± 0.6	2 ± 0.2	1.6 ± 0.1	< LOQ	< LOQ	< LOQ	4.4 ± 0.4	4.3 ± 0.71	n.d.	n.d.	IV
**1,2,3-trideoxy-4,6:5,7-bis-o-[(4-propylphenyl)methylene]-nonitol (NX 8000 K)**	21.8 ± 1.6	23.5 ± 1.1	15.2 ± 4.5	19 ± 2.3	n.d.	n.d.	n.d.	n.d.	32.6 ± 11	39.2 ± 13	27.7 ± 2.3	n.d.	n.d.	n.d.	n.d.	n.d.	n.d.	n.d.	II; SML 5
Octocrylene	28.8 ± 0.3	33.4 ± 1.6	28.2 ± 0.6	22.3 ± 4.9	n.d.	n.d.	n.d.	n.d.	79.5 ± 2.1	503 ± 6	456 ± 33	n.d.	n.d.	n.d.	118 ± 5	122 ± 0.1	n.d.	n.d.	IV; SML 0.05
Avobenzone	< LOQ	< LOQ	< LOQ	< LOQ	n.d.	n.d.	n.d.	n.d.	16.6 ± 0.6	199 ± 12	210 ± 14	n.d.	n.d.	n.d.	30.6 ± 2.6	33.7 ± 0.4	n.d.	n.d.	IV
2-Ethylhexyl 4-methoxycinnamate	47.8 ± 0.2	45.8 ± 0.7	46.7 ± 0.6	49.5 ± 1.6	n.d.	n.d.	n.d.	n.d.	58.2 ± 1.8	198 ± 8	193 ± 8	n.d.	n.d.	n.d.	52.2 ± 0.7	50.3 ± 0.6	n.d.	n.d.	V
**Palmitamide**	< LOQ	< LOQ	< LOQ	< LOQ	n.d.	n.d.	n.d.	n.d.	< LOQ	< LOQ	< LOQ	n.d.	n.d.	n.d.	< LOQ	< LOQ	n.d.	n.d.	IV
2-stearoylglycerol	296 ± 24	247 ± 22	277 ± 1	135 ± 0.4	n.d.	n.d.	n.d.	n.d.	630 ± 29	297 ± 22	262 ± 13	n.d.	n.d.	n.d.	n.d.	n.d.	n.d.	n.d.	II
Chimassorb 81	1.7 ± 0.04	1.2 ± 0.1	1.1 ± 0.1	1.1 ± 0.1	n.d.	n.d.	n.d.	n.d.	1.6 ± 0.1	5.4 ± 0.3	5.2 ± 0.6	n.d.	n.d.	n.d.	n.d.	n.d.	n.d.	n.d.	II; SML 6
**Dioctyl phthalate**	n.d.	n.d.	n.d.	n.d.	n.d.	n.d.	n.d.	n.d.	n.d.	49.2 ± 2.8	57.4 ± 2.1	n.d.	n.d.	n.d.	n.d.	n.d.	n.d.	n.d.	V
**bis(2-ethylhexyl) adipate**	74.6 ± 18	76.9 ± 24	66.4 ± 29	106 ± 1	n.d.	n.d.	n.d.	n.d.	189 ± 8	120 ± 12	79.9 ± 19	n.d.	n.d.	n.d.	31.1 ± 6.4	22.3 ± 1.1	n.d.	n.d.	II; SML 18
Palmitic acid	n.d.	n.d.	n.d.	n.d.	n.d.	n.d.	n.d.	n.d.	n.d.	n.d.	n.d.	n.d.	n.d.	n.d.	n.d.	n.d.	n.d.	n.d.	I; SML 60
**Erucamide**	362 ± 85	279 ± 80	223 ± 86	229 ± 26	n.d.	n.d.	n.d.	n.d.	742 ± 140	917 ± 97	600 ± 271	n.d.	n.d.	n.d.	n.d.	n.d.	n.d.	n.d.	I; SML 60
Irgafos 168	462 ± 151	395 ± 57	365 ± 115	203 ± 200	n.d.	n.d.	n.d.	n.d.	236 ± 98	176 ± 3	374 ± 284	n.d.	n.d.	n.d.	125 ± 38	95.2 ± 0.4	n.d.	n.d.	I; SML 60
**Oleic acid**	n.d.	n.d.	n.d.	n.d.	n.d.	n.d.	n.d.	n.d.	n.d.	n.d.	n.d.	n.d.	n.d.	n.d.	n.d.	n.d.	n.d.	n.d.	I; SML 60
**Docosanamide**	< LOQ	< LOQ	< LOQ	< LOQ	n.d.	n.d.	n.d.	n.d.	< LOQ	< LOQ	< LOQ	n.d.	n.d.	n.d.	n.d.	n.d.	n.d.	n.d.	I; SML 60
**diisodecyl phthalate**	< LOQ	n.d.	n.d.	n.d.	n.d.	n.d.	n.d.	n.d.	< LOQ	6 ± 1.6	4 ± 2.2	n.d.	n.d.	n.d.	n.d.	n.d.	n.d.	n.d.	V
Irganox 1010	210 ± 41	161 ± 18	147 ± 19	89.5 ± 9.3	n.d.	n.d.	n.d.	n.d.	429 ± 32	124 ± 10	195 ± 0.1	n.d.	n.d.	n.d.	184 ± 24	118 ± 35	n.d.	n.d.	I; SML 60
**2,5-Bis(5-tert-butyl-benzoxazol-2-yl)thiophene**	8.8 ± 0.4	6 ± 0.1	6.3 ± 0.7	6.7 ± 0.5	n.d.	n.d.	n.d.	n.d.	9.2 ± 0.3	5.9 ± 0.6	5.5 ± 0.4	n.d.	n.d.	n.d.	7.6 ± 0.7	7.8 ± 0.1	n.d.	n.d.	III; SML 0.6
**Oxidized Irgafos 168**	168 ± 0.1	147 ± 0.1	176 ± 9	208 ± 55	n.d.	n.d.	n.d.	n.d.	348 ± 3	360 ± 10	367 ± 4	n.d.	n.d.	n.d.	198 ± 1	195 ± 4	n.d.	n.d.	IV
Irganox 1076	632 ± 138	549 ± 28	511 ± 17	452 ± 3	n.d.	n.d.	n.d.	n.d.	196 ± 19	153 ± 4	163 ± 6	n.d.	n.d.	n.d.	94 ± 3	107 ± 6	n.d.	n.d.	II; SML 6
Glycerol dihexanoate	n.d.	n.d.	n.d.	n.d.	n.d.	n.d.	n.d.	n.d.	61.2 ± 1.3	52.1 ± 0.7	50 ± 4.7	n.d.	n.d.	n.d.	n.d.	n.d.	n.d.	n.d.	II
